# Expression and Role of Ubiquitin-Specific Peptidases in Osteoblasts

**DOI:** 10.3390/ijms22147746

**Published:** 2021-07-20

**Authors:** Hadla Hariri, René St-Arnaud

**Affiliations:** 1 Research Centre, Shriners Hospital for Children, Montreal, QC H4A 0A9, Canada; hadla.hariri@mail.mcgill.ca; 2 Department of Human Genetics, Faculty of Medicine and Health Sciences, McGill University, Montreal, QC H3A 0C7, Canada; 3 Department of Surgery, Faculty of Medicine and Health Sciences, McGill University, Montreal, QC H3G 1A4, Canada; 4 Department of Medicine, Faculty of Medicine and Health Sciences, McGill University, Montreal, QC H3A 1A1, Canada

**Keywords:** osteoblast, USPs, ubiquitin, bone, skeletal, differentiation, parathyroid hormone

## Abstract

The ubiquitin-proteasome system regulates biological processes in normal and diseased states. Recent investigations have focused on ubiquitin-dependent modifications and their impacts on cellular function, commitment, and differentiation. Ubiquitination is reversed by deubiquitinases, including ubiquitin-specific peptidases (USPs), whose roles have been widely investigated. In this review, we explore recent findings highlighting the regulatory functions of USPs in osteoblasts and providing insight into the molecular mechanisms governing their actions during bone formation. We also give a brief overview of our work on USP53, a target of PTH in osteoblasts and a regulator of mesenchymal cell lineage fate decisions. Emerging evidence addresses questions pertaining to the complex layers of regulation exerted by USPs on osteoblast signaling. We provide a short overview of our and others’ understanding of how USPs modulate osteoblastogenesis. However, further studies using knockout mouse models are needed to fully understand the mechanisms underpinning USPs actions.

## 1. Ubiquitination

Post-translational modification (PTM) of proteins conveys a layer of regulation that affects cellular functions and responses. Ubiquitination, for example, is a complex and dynamic process that has been extensively studied and characterized [1,2]. The proper maintenance of the ubiquitin pool is essential for a multitude of biological events pertaining to cell division and differentiation, signal transduction, and protein degradation by the ubiquitin-proteasome system [3,4]. Perturbations in ubiquitin signaling underlies different pathological conditions including cancer and neurodegenerative disorders such as Alzheimer’s, Huntington’s, and Parkinson’s diseases [5,6,7].

Ubiquitin is a small (76 amino acids) and highly conserved protein among eukaryotes. The life cycle and function of this tiny entity is modulated by classical PTMs such as phosphorylation [8], acetylation [9], adenosine 5′-diphosphate (ADP)-ribosylation [10,11], or modification by ubiquitin-like modifiers such as SUMO, NEDD8, and ISG15 [12]. Ubiquitin can also be ubiquitinated itself on seven different lysine residues (Lys 6, Lys 11, Lys 27, Lys 29, Lys 33, Lys 48, and Lys 63) or on its N-terminal amine group. This key feature allows the addition of ubiquitin to target proteins as a single moiety (monoubiquitin) or as polyubiquitin chains connected by covalent isopeptide bonds. As a result, a permutation of linkages can exist giving rise to branched or mixed polyubiquitin chains that elicit versatile biological outcomes [13,14]. Two well-characterized ubiquitin linkages at Lys 48 and Lys 63 confer this diversity, as Lys 48 polyubiquitination is tied to proteasomal protein degradation, whereas Lys 63-polyubiquitin chains are a signature of signal transduction and DNA repair [1,15]. The complexity of the “ubiquitin code” described by Komander and Rape [1] stems from extensive PTMs and countless possibilities of linkages on ubiquitin entities that dictate the specificity and accessibility to bind protein targets [16,17,18].

During their life span, proteins are ubiquitinated at least once. The ubiquitination process is executed by three different enzyme families: E1 (activation), E2 (conjugation), and E3 (ligation). The enzymatic cascade starts with ATP-dependent activation of ubiquitin catalyzed by E1 enzymes, proceeds with the transfer of activated ubiquitin to the active site of E2 enzymes, and finishes with the ligation of ubiquitin to lysine residues of target proteins by E3 ligases. The human genome comprises hundreds (more than 700) of E3 ubiquitin ligases, enzymes that confer substrate specificity as they selectively bind both E2 enzymes and target proteins.

The act of ubiquitination is reversed by the action of deubiquitinating enzymes (DUBs). Ubiquitin pools are replenished by DUBs and their regulatory roles as proteases are analogous to that of phosphatases in a kinase-phosphatase regulatory scenario. Genome-wide annotation has located nearly 100 putative DUBs across the human genome that cluster into five different families, distinct for their mechanism of catalysis [19]. The DUBs that are cysteine proteases are split into four classes based on their ubiquitin-protease domains: ubiquitin C-terminal hydrolase (UCH), ubiquitin-specific peptidase (USP), otubain protease (OTU), and Machado–Joseph disease protease (MJD). This review focuses on the USP family of deubiquitinating enzymes with particular emphasis on their expression and role in bone-forming cells, osteoblasts.

## 2. USPs: Conserved Structural and Functional Domains

Studies in *Saccharomyces cerevisiae* have led to the discovery and cloning of USP enzymes or UBPs (ubiquitin proteases) in yeast [20]. There exists 16 different *UBP* genes implicated in diverse processes such as: reproduction, energy metabolism, nutrient mobilization, and stress responses [21]. Many USP orthologs have also been identified in the fruit fly *Drosophila melanogaster* [22], the first being fat facets (Faf, ortholog of USP9 in human), a deubiquitinating enzyme implicated in the regulation of Notch signaling in the developing eye [23]. The expansion of the genetic catalogue of USPs across species highlights their significance and conserved multiple functions through evolution [24,25].

The structural annotation of conserved functional domains across species paved the way for the identification of human USP enzymes and USP homologs in other species. In humans, the USP subclass hosts the largest number (58 members) of DUBs. USPs exhibit multiple structural domains that vary in size and architecture; however, they share a high degree of homology within their catalytic domains. The catalytic core of USPs contains three well-conserved motifs: catalytic Cys residue, catalytic His residue, and catalytic Asp/Asn residue [19,25]. This catalytic triad lies within a USP pocket resembling an open hand with a “thumb” (Cys), a “palm” (His/Asp), and “fingers” subdomains that extend to bind ubiquitin molecules [19,25]. Moreover, the size of the catalytic domain can vary between 300 and 800 residues due to large regulatory sequences scattering between the conserved motifs. Additional features of USPs include domains for subcellular localization, substrate specificity, zinc-binding, and ubiquitin recognition [19,25].

The modular feature of USP enzymes grants binding selectivity to both substrates and ubiquitin chains as well as flexibility to switch between active and inactive conformations [26,27]. In theory, the proper binding and catalysis of ubiquitin require an intact catalytic triad that rearranges to position the catalytic cysteine residue in range with the histidine residue [26,27]. On the basis of sequence analysis, six USP proteins (USP39, USP50, USP52, USP53, USP54, and USPL1) are predicted to be devoid of catalytic activity [28]. In this context, the experimental verification of proteolytic activity and biological function is essential. For instance, USP39 (widely known as Sad1p in yeast) lacks all conserved catalytic residues; however, studies have demonstrated a pivotal role for the enzyme in mRNA splicing [29,30] and tumor progression [31].

Given its impact on fundamental biological processes, the interplay among E3-ligases, USPs, and the ubiquitin-proteasome system has been extensively studied [18,32,33,34,35,36,37,38].

## 3. A Glance at the Skeleton

The mineralized skeleton undergoes continuous cycles of remodeling. The coupled activities of bone-resorbing osteoclasts and bone-forming osteoblasts ensure proper bone repair and dynamic adaptation to mechanical stress and microfractures.

Bone remodeling is carried out by an active bone-remodeling unit, involving four major types of bone cells: osteoblasts, osteocytes, bone-lining cells, and osteoclasts. Osteocytes orchestrate bone resorption and bone formation events to maintain normal bone mass. Accumulating evidence suggests that osteocytes can sense bone deformations caused by either mechanical loading or microdamage [39]. Ligand production by osteocytes stimulate the recruitment and activation of osteoclast precursors [39,40] from the general circulation by crossing the bone-lining cell monolayer or from capillaries that irrigate the remodeling unit. In response to high levels of M-CSF and RANKL, osteoclast precursors attach to resorptive sites and differentiate into mature resorbing osteoclasts. This resorptive phase proceeds to dominate the remodeling scene, while the recruitment of osteoprogenitors into the remodeling unit is initiated in the backstage. The recruitment and differentiation of osteoprogenitors into mature osteoblasts continue while resorption is taking place. After this phase, osteoid synthesis by mature osteoblasts becomes the predominant event overtaking bone resorption. This phase allows the remodeling unit to remove more damaged matrix by osteoclasts, while concurrently depositing more osteoid by osteoblasts. Osteoid synthesis continues even after the termination of bone resorption, thus, ensuring a balance between bone resorption and bone formation. Finally, the bone remodeling process is concluded by the mineralization of osteoid deposits, with no net change in bone mass [41]. Maintaining the integrity of the coupling process between osteoblasts and osteoclasts is crucial, in order to avoid skeletal pathologies such as osteoporosis and osteopetrosis [42].

The integrity of the function, differentiation, and crosstalk among the cells in the remodeling unit is crucial for bone homeostasis. Several regulatory mechanisms fueled by transcription factors, ligands, growth factors, hormones, and matrix proteins have been well described [43,44]. The expression of early and late osteoblastogenic differentiation markers dictates the commitment and differentiation of mesenchymal stem cells (MSCs) into osteoblasts. *Runx2* and *Osx* (*Sp7*) are two master regulators of osteogenesis [45,46]. The reciprocal regulation of *Runx2* and *Osx* regulates the proliferation of osteoprogenitors, their commitment to the osteoblast lineage, and osteoblast differentiation. Through differentiation, pro-osteogenic signaling induces the expression of collagenous and noncollagenous bone matrix protein genes such as: type I collagen (*Col1a1*), osteopontin (*Opn*), osteocalcin (*Bglap2*), and matrix gla protein (*Mgp*) [47]. These events and the concomitant production of alkaline phosphatase (*Alpl*) by mature osteoblasts support calcium deposition and proper matrix mineralization.

Landmark advances in ubiquitin biology have unveiled central functions for USPs in regulating the commitment of MSCs and differentiation of osteoblasts [48,49,50] as well as osteoclasts [51,52].

## 4. Regulation of Bone Remodeling

### The Ubiquitin-Proteasome System

Studies have investigated the actions of the ubiquitin-proteasome system during bone remodeling [53]. A pertinent example is the use of bortezomib, a proteasome inhibitor widely used in the clinic to treat multiple myeloma patients [53,54,55]. The hallmark of multiple myeloma is osteolytic bone destruction caused by an imbalanced bone turnover rate. Mechanistically, the administration of bortezomib has been shown to induce osteogenic differentiation of MSCs in mice by regulating RUNX2 [56] and inhibit the degradation of β-catenin [57], Dkk1 [54], and Gli3 [58] to enhance bone formation. More recently, the activation of the IRE1α-XBP1s ER stress signaling has been implicated in bortezomib-induced osteogenesis in vivo [59]. Clinical trials along with in vitro and in vivo myelomatous systems have provided evidence of bortezomib efficacy in inhibiting bone resorption [60]. While different mechanisms have been characterized [60], the targeted inhibition of RANKL-mediated activation of NF-κB in osteoclasts seems to be central for osteoclastogenesis suppression by bortezomib [55,61]. In the context of bone remodeling, bortezomib is one of many other proteasome inhibitors that functions in a similar fashion to combat bone disorders [53].

The adverse off-target effects resulting from the systemic shutdown of the proteasome system pave the way for investigating the functions of other key players in the ubiquitin system including E3-ligases and deubiquitinases during bone remodeling.

## 5. USPs and Osteoblasts

### 5.1. Regulation of Signal Transduction Pathways

Bone morphogenetic proteins (BMPs) and TGFβ are important osteoblastogenic factors, shown to be heavily active during osteogenesis. Among the different BMPs, the pro-osteogenic functions of BMP-2, -4, and -7 have been well documented. The three BMPs are implicated in the regulation of RUNX2-mediated induction of osteoblastogenic markers such as ALP and OCN [62]. The canonical BMP signaling pathway acts through the phosphorylation and nuclear translocation of Smad 1/5/8, leading to enhanced expression of *Runx2*, *Osx*, and *Dlx5* [47]. Moreover, TGFβ binds TGFβRI and TGFβRII receptors and induces osteoblastogenic signaling downstream of Smad2/3 [47]. TGFβ has also been shown to enhance osteoblast proliferation, differentiation, recruitment, and extracellular matrix deposition during bone formation [47]. BMPs and TGFβ also activate noncanonical signaling mediated by TGFβ activated kinase 1 (TAK1) and mitogen-activated protein kinase (MAPK) to induce osteoblast-specific genes [47].

In the past few years, the functional map of USPs in bone expanded considerably. The astounding progress that has been made highlights the role of USPs in modulating osteoblast differentiation and function. Among the different USPs, USP4, USP15, and USP11 are homologs displaying conserved structural and regulatory domains [27]. In addition to homology, the three enzymes were initially discovered as TGFβ receptor-specialized USPs [63,64,65]. 

The TGFβ/BMP signaling pathway is a target of USP4-dependent deubiquitination [35]. Following its phosphorylation and activation by AKT (also known as protein kinase B), USP4 deubiquitinates the TGFβ1 receptor and stabilizes the TGF signal [65], most likely through the Smad7-Smurf2 complex [66]. Moreover, USP4 has been shown to inhibit the monoubiquitination of SMAD4 and enhances BMP signaling in mouse embryonic stem cells [67]. USP4 has also been implicated in the regulation of the non-canonical TGFβ signaling pathway [68]. It deubiquitinates the K63-linked ubiquitin chains of TAK1 leading to the disruption of TNFα- and TGFβ- induction of NF-κβ production [68]. USP4 is also known to regulate the Wnt/β-catenin signaling pathway in osteoblasts. It antagonizes osteoblast differentiation and mineralization through the deubiquitination of Dishevelled (Dvl) and subsequent impairment of Wnt3a-dependent signaling [49]. In metastatic cancer models, USP4 has been shown to deubiquitinate and stabilize β-catenin, thus, enhancing the Wnt signal [69,70]. Taken together, these findings suggest an important role for USP4 in controlling osteogenic differentiation and bone formation signaling.

SMAD7 and ALK5 (TGFβ receptor I), two important effectors of canonical TGFβ signaling, are targets of USP11 enzymatic activity. USP11 deubiquitinates and stabilizes ALK5 to enhance TGFβ-activated transcription and phosphorylation of SMAD2/3 [63]. Bound to SMAD7, USP11 can also augment TGFβ signaling by antagonizing the negative regulatory effect of SMAD7 on ALK5 stability [63]. Consequently, USP11 controls the stability of ALK5 and may have an impact on osteoblast differentiation. 

USP15 is another regulator of TGFβ signaling. It has been reported to enhance TGFβ responses by binding the SMAD7/SMURF2 complex and, subsequently, deubiquitinating ALK5 [64]. Moreover, USP15 is a positive regulator of BMP signaling. USP15 interacts with and deubiquitinates ALK3 (type I BMP receptor), thus, enhancing BMP-mediated phosphorylation of SMAD1 [71]. Apart from TGFβ/BMP signaling, USP15 has been shown to both activate and inhibit Wnt/β-catenin signaling [72]. USP15 enhances bone formation by preventing the ubiquitin-proteasomal degradation of β-catenin through a nonclassical pathway involving FGF2 and MEKK2 in osteoblasts [73]. Concomitantly, USP15 promotes the stabilization of the tumor suppressor protein adenomatous polyposis coli (APC), a critical component of the β-catenin destruction complex and inhibitor of Wnt/β-catenin signaling [74]. These findings highlight an important role for USP15 in regulating osteoblast signaling and bone formation.

The closely related functions of USP4, USP11, and USP15 raise an interesting question as to whether they act independently or in a complex to stabilize type I TGFβ receptors. Further investigation using knockout mouse models for the three USPs or knockin inactive mutants is necessary to answer this question and to define functions pertaining to bone formation and osteoblast function.

### 5.2. Mesenchymal Commitment and Differentiation

Many USPs have been reported to play a role during mesenchymal commitment and differentiation. By handling deubiquitination, USPs control the stability and activity of master protein regulators implicated in cell-lineage fate determination. In this section, we review recent findings highlighting the role of USPs during mesenchymal differentiation with special emphasis on our work related to *Usp53*.

#### 5.2.1. USP34 and USP7

Studies have characterized multiple mechanisms driving the osteogenic differentiation of MSCs. Pertaining to deubiquitinating enzymes, USP34 has been shown to be critical for MSCs differentiation and bone formation [75]. The conditional deletion of *Usp34* in MSCs or pre-osteoblast cells impairs osteoblast differentiation and attenuates BMP2- activated responses. Mechanistically, the loss of USP34 compromises the stability of Smad1 and RUNX2 in vitro [75]. Another regulator of osteogenic differentiation is USP7 [48,50]. A recent study has identified USP7 as a deubiquitinase of RUNX2 in osteoblasts and uncovered the contribution of the CK2/USP7/RUNX2 pathway to both physiological and pathological events of bone formation and mineralization [48]. In addition, USP7 acts through the Wnt/β-catenin arm to regulate osteogenic and adipogenic differentiation [76]. USP7 deubiquitinates and stabilizes Axin, a key scaffold protein important for the assembly of the β-catenin destruction complex, thus, inhibiting Wnt signaling and subsequently modulating differentiation [76].

#### 5.2.2. USP53

The scope of our work covers the characterization of novel molecular mechanisms involved in the anabolic action of parathyroid hormone (1–34) (PTH) in osteoblasts [77,78,79,80]. Administrating PTH at a low dosage once a day (intermittent PTH, iPTH) promotes bone formation through pleiotropic effects on osteoblasts and osteocytes [81,82,83]. The results from numerous studies have shown that multiple signaling pathways act in parallel or synergistically to achieve the full anabolic response to iPTH treatment [84,85]. Work from our laboratory led to the identification and characterization of the nascent-polypeptide-associated complex and coregulator alpha (NACA), a transcriptional coregulator, as a target of the iPTH-PKA axis in osteoblasts (reviewed in [84]). The physiological relevance of this pathway is mediated through the induction of downstream effectors crucial for osteoblast function and bone biology [77,78,79,80]. Using RNA-sequencing and ChIP-sequencing against NACA, we identified *Usp53* as a transcriptional target induced by the iPTH-NACA axis in osteoblasts [77]. The RNA-sequencing data of PTH-treated MC3T3-E1 osteoblastic cells has yielded hundreds of differentially regulated genes [77], some of which belonged to the USP family of deubiquitinases (Table 1). An RNA-seq analysis has revealed that *Usp9x* was predominantly expressed in MC3T3-E1 cells at basal levels, followed by *Usp18*, *Usp12*, *Usp30,* and *Usp36* that showed moderate levels of expression and *Usp53*, *Usp35*, *Usp2*, and *Usp27x* that exhibited the lowest levels of expression [77]. Interestingly, *Usp53* and *Usp2* were significantly upregulated by more than two-fold following iPTH treatment (Table 1). The induction of *Usp53* following PTH treatment was further confirmed by RT-PCR over a time course in calvarial osteoblast cells [77]. Unlike *Usp53*, *Usp2* has been characterized as a target of PTH and has been shown to be crucial for PTH-induced proliferation of osteoblasts in vitro [86]. As for *Usp53*, little is known about its biological function in osteoblasts.

*USP53* fulfills important physiological functions in different tissues [87,88,89,90,91] and has been associated with cancer progression [92,93,94]. Kurban et al. reported a duplication in the genomic locus of *USP53*, *MYOZ2*, and *FABP2* in a patient with bone deformities and severe obesity (BMI > 40), later diagnosed with Cantu syndrome [89]. The identification of *Usp53* as a target of iPTH in osteoblasts raises the question of whether the duplication of *USP53* contributed to the skeletal phenotypic manifestation in this patient. Our recent work on *Usp53* uncovered a role for the gene during mesenchymal differentiation [77]. The knockdown of *Usp53* in mesenchymal cells favored osteoblastogenesis and inhibited adipogenesis in vitro and in vivo [77]. In committed pre-osteoblast MC3T3-E1 cells, the knockdown of *Usp53* enhanced their differentiation potential and increased mineralization [77]. However, these findings have been recently debated by another group. Baek et al. reported a positive role for *USP53* during the osteogenic differentiation of human mesenchymal stem cells (hBMSC) in vitro [95]. One reason for this discrepancy could be the differences between the human (Q70EK8) and the mouse (P15975) protein sequences (72%) that may have led to different protein interactions and function in each species. This has also been supported by the similar phenotypic outcomes observed with *USP53* duplication in human and *Usp53* depletion in murine systems. Another aspect is the ease of overexpression of *Usp53* in human cells [95] and the cytotoxic effect associated with the overexpression of the gene in a multitude of murine cell lines [77]. To sort out this debate, it becomes crucial to investigate the role of *Usp53* in different mesenchymal lineages in vivo, using lineage-specific Cre drivers such as *Prx1*-*Cre*, *Col1a1-Cre*, and *Ocn-Cre*.

**Figure 1:**
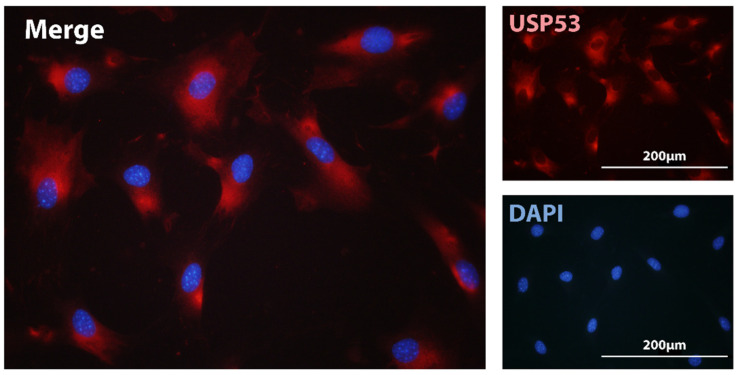
Cytoplasmic localization of USP53 in osteoblast cells. Localization of USP53 in MC3T3-E1 osteoblast cells was examined using immunofluorescence with antibodies against endogenous USP53 (red) and nuclear staining with DAPI (blue). Cytoplasmic localization of USP53 was detected in the merged field. Magnification bars, 200 µm.

USP53 is regarded as an inactive deubiquitinase due to the lack of an essential Histidine residue in its catalytic pocket [27]. Immunofluorescence assays of USP53 in MC3T3-E1 cells localized the protein in the cytoplasm (Figure 1). Devoid of proteolytic activity, we envisage USP53 as a scaffold protein mediating the interaction among different protein partners implicated in mesenchymal commitment and differentiation. This scenario has been supported by the reported interaction of USP53 with ZO-1/TJP1 and ZO-2/TJP2 tight junction scaffolding proteins in epithelial cells of the ear in mice [88]. Recent studies have suggested that USP53 retained some catalytic activity as a deubiquitinase [92,93,95]. Investigating this possibility requires ubiquitination assays to assess catalytic activity. USP53 has been shown to protect β-catenin from degradation through its interaction with FBXO31 ubiquitin ligase in hBMSC [95]. It is, thus, possible that USP53 acts through different mechanisms to control mesenchymal differentiation.

**Figure 2:**
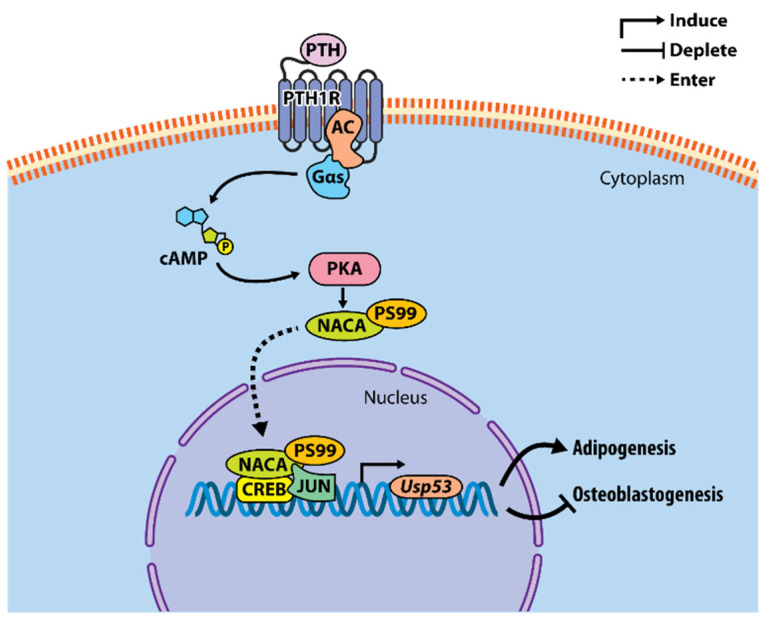
Model of *Usp53* transcriptional regulation and its function in mesenchymal progenitors. PTH binds and activates PTH1R, inducing the coupling of PTH1R to Gαs and activating adenylate cyclase (AC) to produce cAMP and activate protein kinase A (PKA). PKA phosphorylates NACA at serine residue 99 (S99) and induces its nuclear translocation. In the nucleus, NACA binds its response element in the *Usp53* promoter and potentiates transcription of *Usp53* with JUN/CREB in osteoblasts. *Usp53* affects mesenchymal cells lineage decisions, inhibiting osteoblastogenesis and enhancing adipogenesis through mechanisms that remain to be uncovered.

So far, our working model depicts *Usp53* as a transcriptional target of the iPTH-PKA-NACA pathway in osteoblasts. PTH-activated NACA translocates to the nucleus following phosphorylation at serine residue 99 along with CREB and JUN to potentiate the transcription of *Usp53*. The knockdown of *Usp53* in mesenchymal cells enhances osteoblastogenesis and impairs adipogenesis [74]. The mechanism of action by which *Usp53* modulates mesenchymal cell commitment and differentiation is yet to be uncovered (Figure 2).

## 6. Conclusions

Recent studies and emerging evidence keep unfolding the complex layers of control exerted by the ubiquitin/deubiquitinase system. In this review, we have highlighted the functions of USPs in osteoblasts as regulators of signaling outcomes and differentiation decisions. Through specific examples, we have gained insight into the function of USPs in osteoblasts. Further mechanistic studies are pivotal towards developing therapeutic strategies to target USPs and to treat pathological conditions of aberrant ubiquitination.

## 7. Materials and Methods

### 7.1. RNA-seq

RNA-seq was performed as previously described [79]. RNA-seq data were deposited in the Gene Expression Omnibus database of the National Institute for Biotechnology Information (Accession number: GSE154355).

### 7.2. Immunofluorescence

MC3T3-E1 cells were cultured on coverslips, fixed with 3.7% formaldehyde and permeabilized with 0.1% Triton X in PBS. The cells were blocked with 3% BSA for 45 min, and then incubated with rabbit anti-USP53 antibody (HPA035845, Sigma-Aldrich, Oakville, ON, Canada) in PBS with 1% BSA overnight at 4 °C. Then, cells were incubated with anti-rabbit Alexa Fluor–labeled secondary antibodies for 45 min at room temperature, and then mounted with Prolong Gold Antifade containing DAPI (Thermo Fisher Scientific, Waltham, MA, USA). Images were acquired on a Leica DMR fluorescence microscope (Leica Microsystems, Richmond Hill, ON, Canada) connected to a digital DP70 camera (Olympus, Bethlehem, PA, USA).

## Figures and Tables

**Table 1:** Differential regulation of *Usp* genes by PTH (1–34) treatment in MC3T3-E1 cells. RNA-seq analysis of differentially regulated genes following PTH (1–34) treatment in MC3T3-E1 osteoblast cells. A cutoff of 1 in logscale was used to filter regulated genes by a two-fold change with 95% confidence by a moderated *t*-test (*p* < 0.05).

Gene	Log_2_ FC	*p*-Value
*Usp2*	4.65406	5.0 × 10^−5^
*Usp53*	2.17645	5.0 × 10^−5^
*Usp36*	0.851054	5.0 × 10^−5^
*Usp9x*	0.544075	5.0 × 10^−5^
*Usp18*	0.369972	2.9 × 10^−3^
*Usp12*	0.355183	9.5 × 10^−4^
*Usp35*	−1.33667	1.0 × 10^−4^
*Usp27x*	−1.07571	3.0 × 10^−4^
*Usp30*	−0.436837	2.0 × 10^−4^

Log_2_ FC is log_2_ fold change of differentially regulated genes.

## Data Availability

RNA-seq data were deposited in the Gene Expression Omnibus database of the National Institute for Biotechnology Information (Accession number: GSE154355).
